# The relationship of blood CDC42 level with Th1 cells, Th17 cells, inflammation markers, disease risk/activity, and treatment efficacy of rheumatoid arthritis

**DOI:** 10.1007/s11845-021-02858-y

**Published:** 2021-12-02

**Authors:** Yongji Li, Wendi Yang, Feng Wang

**Affiliations:** 1 Department of Rheumatology, The First Affiliated Hospital of Wenzhou Medical University, Wenzhou, 325000 People’s Republic of China; 2 Department of Rheumatology, Xiangyang Central Hospital, Affiliated Hospital of Hubei University of Arts and Science, No. 136 Jingzhou Street, Xiangyang, 441021 People’s Republic of China

**Keywords:** Cell division control protein 42, Disease risk and activity, Rheumatoid arthritis, Th1 and Th17 cells, Treatment efficacy

## Abstract

**Background:**

Cell division control protein 42 (CDC42) is reported to be involved in multiple inflammation processes by regulating T cell differentiation, maintaining immune cell homeostasis, and altering their function, while no relevant studies explored its clinical role in patients with rheumatoid arthritis (RA). Therefore, this study aimed to explore the correlation of CDC42 with Th1 and Th17 cells and its association with disease risk, activity, and treatment outcomes of RA.

**Methods:**

After the enrollment of 95 active RA patients and 50 healthy subjects (HC), their CDC42, Th1 cells, and Th17 cells were assayed by RT-qPCR and flow cytometry, accordingly. For RA patients only, CDC42 was also detected at W6, and W12 after treatment. The treatment response and remission status were evaluated at W12.

**Results:**

Compared to HC, CDC42 was reduced (*P* < 0.001), while Th1 cells (*P* = 0.021) and Th17 cells (*P* < 0.001) were increased in RA patients. Besides, CDC42 was negatively correlated with Th17 cells (*P* < 0.001), erythrocyte sedimentation rate (ESR) (*P* = 0.012), C-reactive protein (*P* = 0.002), and disease activity score in 28 joints (DAS28) (*P* = 0.007), but did not relate to Th1 cells or other disease features (all *P* > 0.05) in RA patients. Furthermore, CDC42 was elevated during treatment in RA patients (*P* < 0.001). Moreover, CDC42 increment at W12 correlated with treatment response (*P* = 0.004). Besides, CDC42 elevation at W0 (*P* = 0.038), W6 (*P* = 0.001), and W12 (*P* < 0.001) also linked with treatment remission.

**Conclusion:**

CDC42 has the potential to serve as a biomarker to monitor disease activity and treatment efficacy in patients with RA.

## Introduction

Rheumatoid arthritis (RA) is a chronic autoimmune disease characterized by systemic inflammation and autoantibodies [1]. Notably, RA places a significant burden on patients since it causes irreversible joint damage that may eventually lead to disability [1, 2]. Although various pharmacological interventions (including non-steroidal anti-inflammatory drugs, conventional synthetic disease-modified anti-rheumatoid drugs (cDMARDs) and biologics etc.) have been applied to manage RA, low treatment remission rate in RA patients has still been reported [3]. Thus, low treatment remission impairs the daily function and even long-term clinical outcomes of RA patients [4, 5]. Recently, several biomarkers (such as certain polymorphisms of B lymphocyte stimulator promoter, autoantibody profile, and anti-drug antibodies etc.) have been identified to predict treatment response in RA patients, which further individualizes RA management and improves their prognosis [6–8].

Cell division control protein 42 (CDC42), a type of small GTPase belonging to the Rho family, has been reported to be involved in regulating actin polymerization and epithelial polarity [9, 10]. In addition, CDC42 also participates in modulating immune response by interacting with neutrophils, T cells, and B cells to maintain their identity, promote cell differentiation, and activate their function [11–14]. For instance, CDC42 suppresses T cell differentiation into T-helper 17 (Th17) cells leading to imbalance of Th17/regulatory T cells (Treg) and development of autoimmunity [12]. Moreover, deletion of CDC42 in naïve T cell promotes T cell differentiation into Th1 cells, CD8^+^ T cells, and memory T cells [14] Furthermore, CDC42 reduces the inflammation characterized by decreased inflammatory cytokine production (such as interferon (IFN)-γ and tumor necrosis factor (TNF)-α) in an animal model of inflammatory bowel disease (IBD) [15]. In the clinical aspect, CDC42 correlates with disease risk and activity of pediatric IBD [16], while no relevant studies have reported the clinical role of CDC42 in patients with RA.

Therefore, the aim of this study was to explore the correlation of CDC42 with RA risk and its association with Th1 cells, Th17 cells, disease activity, and treatment outcomes in patients with RA.

## Methods

### Subjects

This prospective study was approved by the Institutional Research Ethics Committee. From January 2018 to July 2020, a total of 95 active RA patients who were treated in the hospital were consecutively enrolled in the study. The patients who meet the following criteria were eligible for enrollment: (a) diagnosed as RA according to the 2010 American College of Rheumatology criteria [17]; (b) age more than 18 years; (c) disease activity score in 28 joints based on erythrocyte sedimentation rate (DAS28-ESR) more than 3.2; (d) willing to provide peripheral blood (PB) samples for the study use. Patients were excluded if they had the following conditions: (a) presented with active infections; (b) presented with active hepatitis B/C virus; (c) history of cancers or malignancies; (d) joint deformity; (e) during pregnancy or lactation. In addition, during the same period, 50 additional healthy subjects matched for age and gender to RA patients were recruited for the study as health controls (HC). To eliminate the potential sources of bias, the age of HC was limited within 40–70 years old, and the gender ratio of HC was 4:1 (female: male). The written informed consents were obtained from all subjects.

### Collection of data and samples

Clinical characteristics of RA patients were recoded after enrollment, which included demographic characteristics, autoantibodies, and disease activity components. Also, PB samples of RA patients were collected at baseline (W0, then at week 6 (W6) and week 12 (W12) after beginning the treatment, which were used for separation of peripheral blood mononuclear cells (PBMCs) by gradient density centrifugation. For HC, PB samples were also collected after admission to isolate PBMCs.

### Treatment

Depending on the actual disease situation, the patients received corresponding treatment. Seventy-four patients received monotherapy or combination treatment with conventional disease-modifying antirheumatic drug (cDMARD), while 21 patients received biologics-based regimen, including tumor necrosis factor inhibitor (TNFi) and interleukin-6 inhibitor (IL-6i), with or without cDMARD combination.

### Determination of CDC42 expression

PBMCs of all subjects were used to assess the expression of CDC42 by reverse transcription quantitative polymerase chain reaction (RT-qPCR)*.* In brief, the total RNA was extracted from PBMCs using the QIAamp RNA Blood Mini Kit (Qiagen, Duesseldorf, Nordrhein-Westfalen, Germany). Then, reverse transcription was achieved using PrimeScript™ RT reagent Kit (Perfect Real Time) (Takara, Dalian, Liaoning, China). Subsequently, the PCR reaction utilized QuantiNova SYBR Green PCR Kit (Qiagen, Duesseldorf, Nordrhein-Westfalen, Germany). The relative expression of CDC42 was calculated by the 2^−ΔΔCt^ method using GAPDH as an internal reference. The detailed sequences of forward and reverse primers for CDC42 and GAPDH were in line with a previous study [18].

### Determination of Th1 and Th17 cells

Th1 and Th17 cells (within CD4^+^ T cells, %) were determined by multicolor flow cytometric analysis using the HumanTh1/Th17 Phenotyping Kit (Becton, Dickinson and Company, Franklin Lake, NJ, USA). The experiment was carried out referring to complete procedure of the kit specification. Briefly, T helper cells were stimulated by phorbol myristate acetate (PMA) plus Ionomycin. Afterwards, the cells were transferred into flow rest tube for immunofluorescent staining with specific fluorescent antibodies. Then, the stained cells were analyzed by flow cytometric analysis using a fluorescence activated cell sorting (FACS) flow cytometer. Finally, Th1 and Th17 cells (within CD4^+^ T cells, %) were calculated.

### Assessment of complete response and complete remission

According to the European League Against Rheumatism (EULAR) response criteria [19], complete response and complete remission were assessed at W6 and W12 after initiation of treatment, based on DAS28-ESR score, which was calculated by tender joint count (TJC), swollen joint count (SJC), and ESR, and the formula was as follows: 

$$DAS28-ESR=\lbrack0.56\sqrt{TJC}+0.28\sqrt{SJC}+0.70\ast\ln(ESR)\rbrack\ast1.08+0.16$$

Complete response was defined as a DAS28-ESR score decreased more than 1.2 from baseline, and complete remission was defined as a DAS28-ESR score of less than 2.6. The RA patients were classified as response and non-response as well as remission and non-remission, based on W12 response and remission status.

### Statistical analysis

Statistical analysis and graph plotting were completed using SPSS 24.0 (IBM Corp., Armonk, NY, USA) and GraphPad Prism 6.01 software (GraphPad Software Inc., San Diego, CA, USA), respectively. A total of 9 (9.5%) patients lost follow-up during the study, and all of them had at least one follow-up record. The last observation carried forward (LOCF) method was applied to process the missing data, and all patients were included in the analysis. Comparison of CDC42 expression, Th1 cells (within CD4^+^ T cells, %), and Th17 cells (within CD4^+^ T cells, %) between two groups was assessed by Mann–Whitney *U* test, and receiver operating characteristic (ROC) curve was used for estimating their profiles in distinguishing different subjects. Correlation between two variables was analyzed by Spearman’s rank correlation test. Changes of CDC42 expression over time were determined by Friedman test, and comparisons of CDC42 expression between response and non-response patients as well as remission and non-remission patients were determined by Mann–Whitney *U* test. Statistical significance was concluded if there was a *P* value < 0.05 in the above analysis.

## Results

### RA patients’ clinical features

RA patients presented with a mean age of 54.8 ± 9.8 years, consisting of 77 (81.1%) females and 18 (18.9%) males. The mean disease duration of RA patients was 3.5 ± 3.3 years. In terms of autoantibodies, 76 (80.0%) RA patients were positive for rheumatoid factor (RF) and 56 (58.9%) RA patients were positive for anti-cyclic citrullinated peptide antibody (ACPA). Moreover, the mean DAS28-ESR score of RA patients was 5.0 ± 0.7. There were 79 (83.2%) RA patients who received methotrexate (MTX) treatment prior to our study. Regarding the treatment, 21 (22.1%) RA patients received biologics-based regimen, and 74 (77.9%) RA patients received cDMARDs therapy. Moreover, the detailed clinical features of RA patients are listed in Table 1.

**Table 1:** Clinical features of RA patients

Items	RA patients (*N* = 95)
**Demographics**	
Age (years), mean ± SD	54.8 ± 9.8
Gender, No. (%)	
Female	77 (81.1)
Male	18 (18.9)
BMI (kg/m^2^), mean ± SD	22.4 ± 2.8
Disease duration (years), mean ± SD	3.5 ± 3.3
**Autoantibodies**	
RF, No. (%)	
Positive	76 (80.0)
Negative	19 (20.0)
ACPA, No. (%)	
Positive	56 (58.9)
Negative	39 (41.1)
**Disease activity components**	
TJC, median (IQR)	6.0 (5.0–8.0)
SJC, median (IQR)	5.0 (3.0–7.0)
ESR (mm/h), median (IQR)	31.0 (23.2–45.1)
CRP (mg/L), median (IQR)	23.5 (11.2–42.5)
DAS28-ESR score, mean ± SD	5.0 ± 0.7
HAQ-DI score, mean ± SD	1.2 ± 0.3
**Treatment**	
History of MTX treatment, No. (%)	79 (83.2)
Biologics-based regimen (TNFi or IL-6i), No. (%)	21 (22.1)
cDMARD (monotherapy or combination), No. (%)	74 (77.9)

*RA* rheumatoid arthritis, *SD* standard deviation, *BMI* body mass index, *RF* rheumatoid factor, *ACPA* anti-cyclic citrullinated peptide antibody, *TJC* tender joint count, *IQR* interquartile range, *SJC* swollen joint count, *ESR* erythrocyte sedimentation rate, *CRP* C-reactive protein, *DAS28-ESR* disease activity score in 28 joints based on erythrocyte sedimentation rate, *HAQ-DI* health assessment questionnaire disability index, *MTX* methotrexate, *TNFi* tumor necrosis factor inhibitor, *IL-6i* interleukin-6 inhibitor, *cDMARD* conventional disease-modifying antirheumatic drug

### CDC42, Th1 cells, and Th17 cells in RA patients and HC

**Fig. 1:**
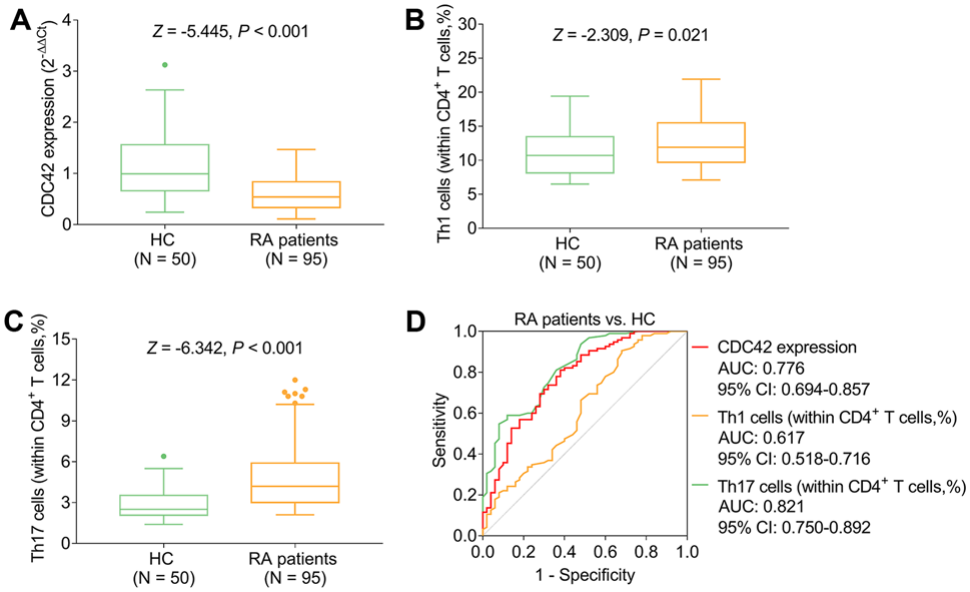
Comparison of CDC42, Th1 cells and Th17 cells between RA patients and HC. Comparison of CDC42 (**A**), Th1 cells (**B**), and Th17 cells (**C**) between RA patients and HC. Correlation of CDC42, Th1 cells, and Th17 cells with RA risk (**D**). CDC42, cell division control protein 42; RA, rheumatoid arthritis; Th1, T-helper 1; Th17, T-helper 17; HC, healthy control

Compared to HC, CDC42 was reduced (*P* < 0.001, Fig. 1A), whereas Th1 cells (*P* = 0.021, Fig. 1B) and Th17 cells (*P* < 0.001, Fig. 1C) were increased in RA patients. Further ROC curve analyses showed that Th17 cells (area under curve (AUC): 0.821, 95% confidence interval (CI): 0.750–0.892), CDC42 (AUC: 0.776, 95% CI: 0.694–0.857), and Th1 cells (AUC: 0.617, 95% CI: 0.518–0.716) all could distinguish RA patients from HC (Fig. 1D).

### Correlation of CDC42 with Th1 cells and Th17 cells

**Fig. 2:**
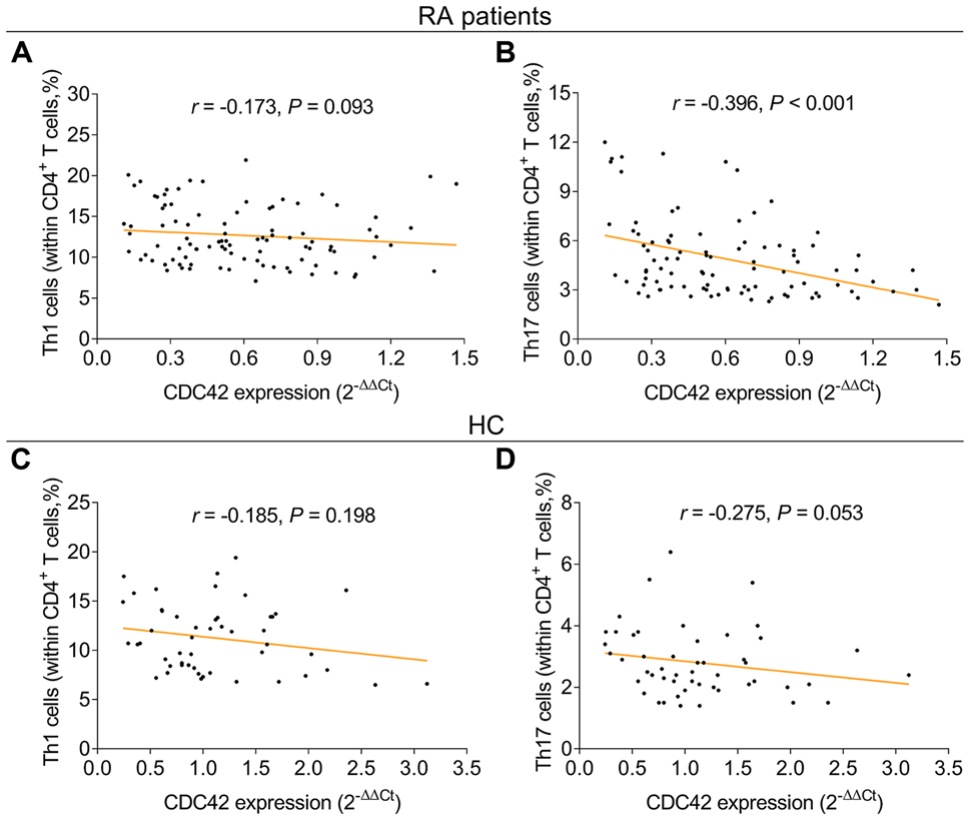
CDC42 was negatively correlated with Th17 cells in RA patients. Correlation of CDC42 with Th1 cells (**A**) and Th17 cells (**B**) in RA patients. Correlation of CDC42 with Th1 cells (**C**) and Th17 cells (**D**) in HC. CDC42, cell division control protein 42; RA, rheumatoid arthritis; Th1, T-helper 1; Th17, T-helper 17; HC, healthy control

Although CDC42 was not correlated with Th1 cells (*r* =  −0.173, *P* = 0.093, Fig. 2A), it was negatively correlated with Th17 cells (*r* =  −0.396, *P* < 0.001, Fig. 2B) in RA patients. However, no correlation of CDC42 with Th1 cells (*r* =  −0.185, *P* = 0.198, Fig. 2C) or Th17 cells (*r* =  −0.275, *P* = 0.053, Fig. 2D) in HC was observed.

### Correlation of CDC42 with clinical features

**Fig. 3:**
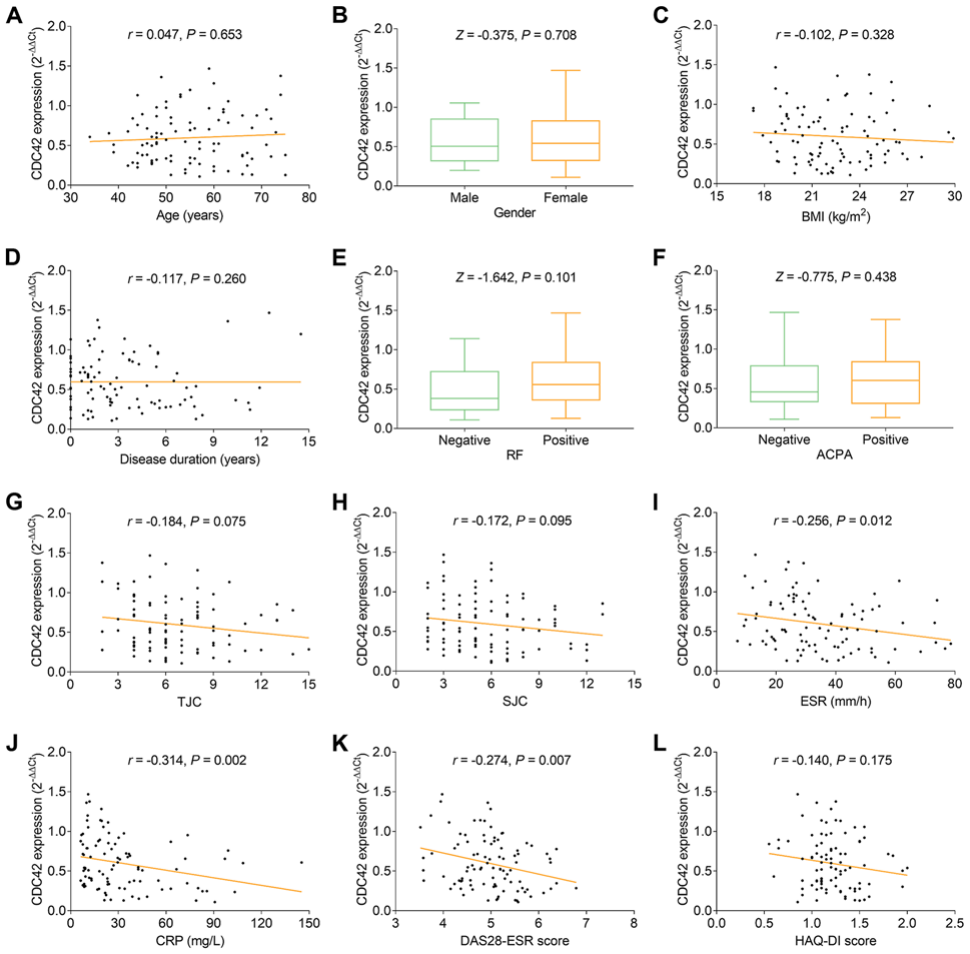
CDC42 was negatively correlated with ESR, CRP, and DAS28-ESR score in RA patients. Correlation of CDC42 with age (**A**), gender (**B**), BMI (**C**), disease duration (**D**), RF status (**E**), ACPA status (**F**), TJC (**G**), SJC (**H**), ESR (**I**), CRP (**J**), DAS28-ESR score (**K**), and HAQ-DI score (**L**) in RA patients. CDC42, cell division control protein 42; RA, rheumatoid arthritis; BMI, body mass index; RF, rheumatoid factor; ACPA, anti-cyclic citrullinated peptide antibody; TJC, tender joint count; SJC, swollen joint count; ESR, erythrocyte sedimentation rate; DAS28-ESR, disease activity score in 28 joints based on erythrocyte sedimentation rate; HAQ-DI, health assessment questionnaire disability index

There was no correlation of CDC42 with age, gender, BMI, disease duration, RF status, ACPA status, TJC, SJC, or HAQ-DI score in RA patents (all *P* > 0.050, Fig. 3A–H, L), while CDC42 was negatively correlated with ESR (*r* =  −0.256, *P* = 0.012, Fig. 3I), CRP (*r* =  −0.314, *P* = 0.002, Fig. 3J), and DAS28-ESR score (*r* =  −0.274, *P* = 0.007, Fig. 3K).

### Correlation of CDC42 longitudinal change with treatment efficacy

**Fig. 4:**
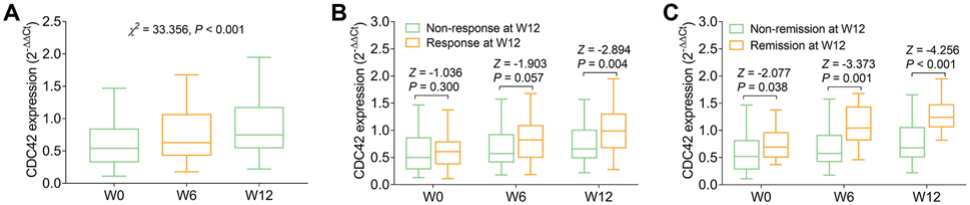
Elevated CDC42 during treatment related to treatment efficacy. Longitudinal change of CDC42 during treatment in RA patients (**A**). Correlation of CDC42 longitudinal change with treatment response at W12 (**B**) and treatment remission at W12 (**C**) in RA patients. CDC42, cell division control protein 42; RA, rheumatoid arthritis; W, week

CDC42 was elevated during treatment in RA patients (*P* < 0.001, Fig. 4A). Further subgroup analyses exhibited that CDC42 at W12 was increased in RA patients who experienced response at W12 compared to those who did not at W12 (*P* = 0.004, Fig. 4B). Moreover, CDC42 at W0 (*P* = 0.038, Fig. 4C), CDC42 at W6 (*P* = 0.001), and CDC42 at W12 (*P* < 0.001) were all higher in RA patients who achieved remission at W12 than those who did not achieve remission at W12.

## Discussion

CDC42, as a small GTPase, plays a critical role in intracellular signaling pathways, particularly in the regulation of actin cytoskeleton dynamics, cell polarity, and cell morphology [20]. Apart from its normal biological function, CDC42 has been reported being involved in the regulation of immune response, osteoclast formation, and myeloid lineage commitment [11–13, 21, 22]. In the clinical field, few studies have reported the clinical value of CDC42 in inflammation-mediate diseases, while only one study reported that CDC42 expression was reduced in activate IBD pediatric patients compared to controls; also it correlated with activate status of pediatric IBD [16]. However, the clinical role of CDC42 in RA patients remains unclear. Therefore, this study was conducted and we discovered that CDC42 was reduced, while Th1 and Th17 cells were increased in RA patients compared to controls. Moreover, CDC42, Th1 cells, and Th17 cells could distinguish RA patients from controls. The possible reasons to explain these findings were as follows: (a) CDC42 suppressed the immune response via regulating T cell receptor and interleukin (IL)-7 receptor signaling pathways, which led to a reduction in systemic inflammation and lower RA risk [15, 23]. (b) Th1 and Th17 cell promoted inflammatory cytokine production (such as IFN-γ, IL-17, and IL-23), which led to elevated systematic inflammation and RA risk [24, 25].

Apart from the aberrant expression of CDC42 in inflammation-mediated diseases, its correlation with disease activity is also of great interest. For instance, CDCD42 was negatively correlated with disease activity reflected by pediatric Crohn’s disease activity index in pediatric IBD patients [16], although no relevant studies have reported the correlation of CDC42 with disease activity or inflammation in RA patients. In the present study, we revealed that CDC42 was negatively correlated with Th17 cells, ESR, CRP, and DAS28-ESR score in RA patients, which could be explained by the following. (a) CDC42 inhibited the differentiation of regulator T cell into Th17 cells, which resulted in a negative correlation between CDC42 and Th17 cells in RA patients [12]. (b) CDC42 suppressed immune response through multiple mechanisms (such as maintenance of T cell identity and regulation of T cell activation etc.) as discussed earlier, which thereby led to reduced inflammation and disease activity in RA patients [11–13].

The identification of potential biomarkers to monitor disease progression and treatment efficacy is necessary for RA patients’ management, while there are no relevant studies reporting the correlation of CDC42 with treatment efficacy in RA patients. In order to explore the longitudinal change in CDC42 and its correlation with treatment efficacy in RA patients, multiple-time measurements (at W0, W6, and W12 after the initiation of treatment) of CDC42 were conducted in our study. Then, we observed an increase of CDC42 during treatment in RA patients. Also, the increment of CDC42 correlated with better treatment efficacy in RA patients, which could be explained by the following: elevated CDC42 was correlated with reduced systematic inflammation as mentioned earlier, which thereby led to better treatment efficacy in RA patients [12, 15].

Several issues were needed to be discussed. For instance, we recruited 95 RA patients and 50 HC since the main purpose of this study was to observe a longitudinal change of CDC42 level and its correlation with treatment response in RA patients, instead of the comparison of CDC42 level between RA patients and HC. Secondly, CDC42 regulated T cell differentiation into Th1 cells and Th17 cells, while it had little effect on regulating Th2 cells in autoimmune diseases; thus, we only detected Th1 cells, Th17 cells, and their secreted cytokines in RA patients instead of Th2 cells in the current study [12, 14]. Thirdly, CDC42 regulated immune response by interacting with neutrophils, B cells, and T cells in the bloodstream; therefore, high blood CDC42 might influence the chronic inflammation and proliferative status of synovial joint in RA patients, while this hypothesis needed further validation [11–13]. Fourthly, limited study reported the clinical value of CDC42 in immune diseases; thus, more attention should be paid to explore the correlation of CDC42 with disease risk and activity in patients with immune diseases. Finally, combined cDMARDs or biologics-based regimen was applied in treating RA patients who failed to respond to single cDMARD in the current study.

However, there were some limitations in the present study. For instance, the sample size in the present study was relatively small, where further study with a larger sample size to validate these findings was necessary. Moreover, we only detected the CDC42 level from PBMC, while its level from other biological samples (such as synovial tissue, serum, exosome etc.) in RA patients was not determined. Furthermore, the age-/gender-matched disease control (such as patients with osteoarthritis) was not recruited in the current study where a comprehensive analysis of CDC42 expression among RA patients, disease control, and HC was needed.

In conclusion, CDC42 was reduced and negatively correlates with Th17 cells, inflammation, and disease activity in RA patients. Moreover, CDC42 was elevated during treatment and its increment correlates with better treatment outcomes in RA patients, indicating its potential clinical value for RA management.
